# Emergence of structure in mouse embryos: Structural Entropy morphometry applied to digital models of embryonic anatomy

**DOI:** 10.1111/joa.13031

**Published:** 2019-07-05

**Authors:** William Waites, Jamie A. Davies

**Affiliations:** ^1^ School of Informatics University of Edinburgh Edinburgh UK; ^2^ Deanery of Biomedical Sciences University of Edinburgh Edinburgh UK

**Keywords:** developmental anatomy, graph theory, information theory, metrics, morphometry

## Abstract

We apply an information‐theoretic measure to anatomical models of the Edinburgh Mouse Atlas Project. Our goal is to quantify the anatomical complexity of the embryo and to understand how this quantity changes as the organism develops through time. Our measure, *Structural Entropy*, takes into account the geometrical character of the intermingling of tissue types in the embryo. It does this by a mathematical process that effectively imagines a point‐like explorer that starts at an arbitrary place in the 3D structure of the embryo and takes a random path through the embryo, recording the sequence of tissues through which it passes. Consideration of a large number of such paths yields a probability distribution of paths making connections between specific tissue types, and *Structural Entropy* is calculated from this (mathematical details are given in the main text). We find that Structural Entropy generally decreases (order increases) almost linearly throughout developmental time (4–18 days). There is one `blip’ of increased Structural Entropy across days 7–8: this corresponds to gastrulation. Our results highlight the potential for mathematical techniques to provide insight into the development of anatomical structure, and also the need for further sources of accurate 3D anatomical data to support analyses of this kind.

## Introduction

In his important text on developmental biology (Kauffman, 1993), Kauffman argues that order in living creatures arises from a combination of evolution and self‐organisation. A remarkable fact about this beautiful text is that the meaning of its title, ‘Origins of Order’ is left essentially implicit: the meaning of the word ‘order’ is never defined. It is discussed extensively, contrasted with ‘chaos’, and asserted as a property of various remarkable observations about fitness landscapes, but we may continue to wonder what, precisely, is meant by ‘order’. It is not hard to account for this ambiguity; exactly what should be meant by order, or related words such as structure or complexity as they apply to biological organisms, is not at all obvious. Indeed, authors such as Grizzi & Chiriva‐Internati (2005) consider the meaning of anatomical structure in detail, making the key point that ‘complexity can reside in the *structure* of the system,’ and suggest the use of mathematics to quantify this, without explaining precisely how.

In this paper, we offer a possibility for quantifying a particular kind of order: the physical structure that develops as an organism grows. We call this measure *Structural Entropy*.

Structural Entropy is a quantity calculated on an abstract representation of the organism's anatomy. To understand how Structural Entropy works, it is helpful to consider the general concept of entropy in Information Theory (Shannon, 2001). The quantity now known in that field as entropy was originally called ‘uncertainty’ by Shannon. Given a probability distribution over some set, if the set is dominated by many equally likely elements (as in a normal pack of cards), the outcome of choosing one at random is very unpredictable, and hence the entropy will be large. If some elements are much more likely to be chosen than others (e.g. in a pack of cards containing 50 jokers and two aces of spades), we can be a bit more certain about the outcome and the entropy will be smaller. In this report, we use this concept, as well as our previous work (Waites et al., 2018) to construct such a probability distribution using the topology of an anatomical model, augmented with geometrical data. This distribution says how likely it is to find a notional particle, allowed to travel freely through the embryo, in any given embryonic tissue.

There is, however, a major obstacle in applying this measure, especially in developmental anatomy: the lack of sufficient good quality data to support applications of the type we propose. What is required is a complete library of accurate digital models (‘atlases’) of embryonic anatomy, from closely spaced stages of development, each digitally annotated so that each pixel (2D) or voxel (3D) is labelled with the identity of the tissue in which it lies. We will refer to this labelling process as ‘tagging’. The best current approximation of such a data library is the Edinburgh Mouse Atlas, or eMouseAtlas (Davidson et al., 2001; Baldock et al., 2003; Christiansen et al., 2006; Richardson et al., 2009, 2013; Armit et al., 2012, 2015). The eMouseAtlas was constructed by digitisation of serial sections of complete mouse embryos at closely spaced stages of development. The different tissues in each digital image were identified and delineated by expert embryologists, who tagged the different regions of the embryos with the tissue identity. These tagged images were then assembled into 3D models of the corresponding embryo, and the datasets are available online. We use the eMouseAtlas to illustrate how Structural Entropy can be calculated and show that it captures structure increasing with time. However, there are very few datasets of this kind available.

The eMouseAtlas contains 3D tagged anatomical models of house mouse (*Mus musculus*) embryos at a selection of pre‐natal stages of development. It is the best freely available dataset of its kind for demonstrating the kind of analysis that we suggest. Nevertheless, it has some defects and inconsistencies which we detail in the section ‘Mouse Atlas’. More broadly, good quality 3D tagged anatomical models for every developmental stage are simply not available for any organism. The similarly named Worm Atlas (Altun et al., [Link]), which uses the model organism *Caenorhabditis elegans*, contains a wealth of resources: diagrams of adult organisms, cell lineages and gene expression data, but only scattered anatomical models. There is a wealth of magnetic resonance image data available for the human brain (Van Essen et al., 2013), but this is intentionally distributed in a minimally processed way to encourage development of techniques for identifying structures within images and further processing. These data are therefore not immediately amenable to the analysis that we advocate here, though it is possible to imagine intermediate processing of those images that could make it so.

Much previous work on the complexity of models of anatomical features is from neuroscience. Several authors characterise complexity as a dynamic quantity. Tononi et al. (1994) introduced an information‐theoretic measure called *Neural Complexity (NC)*. They measure the temporal patterns of signals through neural networks and claim (also Sporns et al., 2000) that these patterns must depend strongly on the underlying anatomical structure. Later authors such as Fan et al. (2017) consider information‐theoretic measures on the neural connectome directly. Horn et al. (2014) use a random‐walk approach at a much finer grain to find agreement between the structural and functional connectivity for the brain's default‐mode network. Chan et al. (2014) use this technique to measure desegregation of brain networks with age and long‐term memory function. For practical reasons, suitable data pertaining to human developmental anatomy is difficult to obtain (Huang et al., 2006; Mietchen & Gaser, 2009), particularly for early developmental stages; therefore computational morphometry is applied mainly to the study of diseases related to ageing (Testa et al., 2004; Matsuda, 2013). We believe that our Structural Entropy measure might also provide a useful diagnostic signal in the context of this kind of ageing study and suggest this as an area of future research.

One of us (Davies, 2016) considered a similar question to that which concerns us here, using a different subset of the eMouseAtlas data. Davies considered the text annotations, and the number of terms required to describe each developmental stage, arguing that the greater number of terms needed, the greater the complexity. From these data, Davies showed that the number of vocabulary terms increases exponentially over time. We show here that what Davies’ result provides is, in fact, a lower bound on order. In this article we confine ourselves to developing Structural Entropy in the context of the data from the eMouseAtlas and show that it captures something of the intuitive idea of increasing anatomical order as development progresses. This line of reasoning relies on the assumption that the anatomical analysis is a faithful representation of the underlying structure in the organism. We show that Structural Entropy appears reasonably robust to inconsistencies in manual analysis and tagging.

## Methods

Because the detailed mathematical description of our methods (section ‘Technical description’) may not be easily accessible to all readers, we provide an additional illustrated description written in non‐technical English. This account (section ‘Informal description of method’) captures the essence of how our analysis works but necessarily involves informal and imprecise analogies; readers wishing to criticise, replicate or build on our work are strongly advised to engage directly with ‘Technical description’.

### Informal description of method

As mentioned in the introduction, our concept of Structural Entropy is related to Claude Shannon's concept of ‘uncertainty’ (later called ‘entropy’) in the field of Information Theory. This is a measure of disorder, or unpredictability, in a set of data. If the outcome of a random dip into a bag of data elements is known with high probability (e.g. if 90% of the numbers in the dataset were ‘1’), then the predictability would be high and the entropy low. If the outcome of the random dip were only known with very low probability (e.g. the numbers in the dataset were truly random), then the predictability would be low and the entropy high.

**Figure 1:**
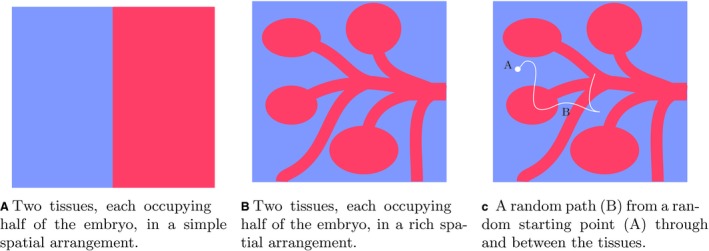
Tissues in simple and rich spatial arrangements and an example random path.

The structure of an embryo, or any other biological object, can be modelled as a bag of data, each data element comprising 3D coordinates (*x*, *y*, *z*) that specify its position and a tag that specifies the tissue name at that point. A naive approach to measuring the degree of order might therefore be to make many random dips into the dataset for an embryo, and calculate the probability distribution of finding a tag for different tissues (e.g. ‘ectoderm’, ‘mesoderm’, etc. for a gastrulation‐stage embryo), and use this to make a measure of structure. This approach, however, has a serious problem: an embryo that consisted of two tissues each of which occupied one half of the embryo (Fig. 1A) would have the same probability distribution as one that consisted of the same 50/50 mix of two tissues in a rich spatial arrangement (Fig. 1B). Clearly, a measurement that would ignore such rich anatomical organisation would not be useful.

To avoid this problem, we consider not simply random dips into embryological data, but random paths taken through the embryo. We begin at a random point and allow a particle to traverse a random path (Fig. 1c). Then, after doing this for many starting points and paths, we can calculate the probability distribution that a path starting in tissue 1 (say, ectoderm) finishes in tissue 2 (say, endoderm) within a certain number of steps. It can be seen intuitively that the probability distributions that would result from the anatomy in Fig. 1A, where most short paths would never leave their starting tissue, would be very different from those resulting from the anatomy in Fig. 1B. This way of proceeding does, therefore, capture a measure of anatomical richness as well as simple proportions of composition.

We use these path‐based probability distributions to calculate Structural Entropy, as defined in section on ‘Structural Entropy’ below. This involves one important adjustment. Clearly, the more different tissues there are in an embryo, the more alternatives there are for the tissue‐type tag corresponding to a spatial position, and the higher the maximum entropy. To avoid our measure being dominated by this trivial effect, we calculate the maximum possible entropy (highest possible disorder) of each embryonic stage by imagining all its tissues being present in an arbitrarily fine, random jumble. We then divide our measure of Structural Entropy from that embryo by the maximum possible entropy, to provide a normalised measure of Structural Entropy that can be compared, fairly, between different embryonic stages that contain different numbers of tissues.

### Technical description of method

#### Path entropy

We previously defined Path Entropy as a measure of patterning on tagged graphs (Waites et al., 2018) and we give a brief summary here. See Glossary for the meaning of ‘graph’ and ‘tagged’ in this context. In our original treatment (Waites et al., 2018) we used the word ‘colour’ instead of tag, as is usual in computer science.

The intuition underlying Path Entropy is as follows. The standard notion of entropy for 2D images is constructed from the probability distribution of pixel colour values (Mangin, 2000; Gonzalez et al., 2004; Tsai et al., 2008). The probability of a pixel being green, say, is just the fraction of pixels that are green. To capture more structure, we generalised it in two ways. First, rather than a regular rectangular lattice as in a digital image, we allow an arbitrary graph, with each vertex having a tag. Secondly, we consider not only the probability of a vertex having a given tag, but the conditional probability distribution of its neighbours’ tags. This is then extended to neighbours’ neighbours and so forth, for paths of a given length. More formally, let *G* = (*V*, *E*, *C*, *χ*) be a tagged graph, where *V* and *E* are vertices and edges (see Glossary), *C* is a set of tags, and *χ* is a function that gives the tag corresponding to a vertex. In other words if *v* is a vertex in this graph, then *χ*(*v*) is its colour. This is enough to re‐create the standard image entropy mentioned above by counting the number of vertices with tag *a* and dividing by the total number of vertices,

(1) $$p\left(a\right)=\frac{\left|v\in V,\chi (v)=a\right|}{\left|V\right|}$$

After all, a pixel grid can be thought of as a graph where each pixel is a vertex and pixels are adjacent if they share an edge.

Instead of considering the vertices on their own, consider now how they are connected together. A path in the graph is a sequence of vertices connected by edges (loops are allowed). A path of length *n* is a sequence of *n* + 1 vertices connected by *n* edges in the graph. Define the function *χ*_*n*_ to be the analogue of *χ*: rather than giving the tag for a single vertex, *χ*_*n*_(*σ*) gives the sequence of tags corresponding to a sequence of vertices *σ*. If we call *S*_*n*_ the set of all paths of length *n* in the graph, then we can find the probability of a tag sequences *s* by analogously counting all of the paths that have that sequence,

(2) $$p_{n}\left(s\right)=\frac{\left|\{\sigma \in S_{n},\chi_{n}\left(\sigma \right)=s\}\right|}{|S_{n}|}$$

The *n*th order Path Entropy is then defined simply as the entropy of this distribution,

(3) $$E_{n}=-\sum_{s\in C^{n+1}}p_{n}\left(s\right)\log\left(p_{n}\left(s\right)\right)$$

#### Structural Entropy

A 3D anatomical model is not an abstract graph with edges and indistinguishable vertices. It consists of regions in space that have particular shapes, each region has a certain tag, and regions can be adjacent to each other. To extend Path Entropy to a setting where it can be applied to regions with spatial extent, accounting for their geometrical structure, we reason as follows.

Begin with a space, *X*, with a Lebesque measure. In two or three dimensions, this corresponds to normal Euclidean space, but for generality we are not concerned so long as length, area, volume and any higher‐dimensional analogous concepts are well‐defined and can be summed or integrated over. Let this space be sub‐divided in to a set of regions, $R=\left\{R_{i}\right\}$, and ask what the probability is, if a point is chosen uniformly at random, that it will be found in a given region, *R*_*i*_. This probability, is the fraction of the total volume occupied by that region,

(4) $$p\left(R_{i}\right)=\frac{\int_{R_{i}}dx}{\sum_{j}\int_{R_{j}}dx}$$

Analogously to the discrete case of image entropy, define the function *χ* to yield the tag for a given region. We can find the probability of a certain tag, *c*, by adding up the probabilities of choosing a point in a region with that tag,

(5) $$p_{c}=\sum_{\left\{R_{i}\in R,\chi \left(R_{i}\right)=c\right\}}p\left(R_{i}\right)$$

We would like to extend this in a way that accounts for the shape of the regions and their adjacencies with each other. To provide some intuition to guide us, we use the idea that structure is related to communication. In a living organism, the shapes that different anatomical systems have are strongly influenced by communication. Nutrients and chemical signals travel along physical pathways and diffuse across boundaries. The travel of these molecules from one system to another (possibly undergoing transformation along the way) is a kind of communication. Exchange of molecules between systems is facilitated by relatively larger shared boundaries. This constraint influences the shape of the system. Minimising boundary size results in a spherical shape, so the degree to which diffusion and hence communication is prioritised is the degree to which the volume occupied by the system differs in shape from a sphere.

Proceeding on this basis, imagine that the randomly chosen point somewhere, in some region, is a notional molecule or particle. This particle is allowed to drift randomly in each region. When it comes to the edge of a region adjacent to another it may diffuse across this boundary. After some time, the particle will be found in some region, possibly having traversed some others. If *s*_1_ represents the path taken through the first region, *s*_2_ the path taken through the second, and so forth, the sequence, *s*_1_, …, *s*_*n*_, represents the trajectory of the particle. There is a tag that corresponds to each region, so there is a tag sequence that corresponds to this trajectory. If we can work out from the data all of the tag sequences that can be produced by the notional wandering particle, then we can ask, as we did before (Eq. (2)) for a probability distribution of tag sequences. We call the entropy of this distribution the *Structural Entropy*.

One way to work out the distribution of tag sequences is to consider all the possible paths that this particle might take through the various regions from each starting, to each ending point. This approach affords a large degree of flexibility for modelling: each region can contribute in different ways to the action, encoding more information than is present in the spatial relations themselves. However, the data necessary for such an ambitious approach are not available and it is far from clear how to model appropriately the contributions of different anatomical regions to the complexity of the organisms as a whole.

We restrict the question to what can be answered with the available data. To this end, we ask instead, given that the particle started in a region with the tag *c*_*i*_‐, what is the chance that it eventually ends up in one with the tag *c*_*j*_? This question allows us to quantify the notion of communication or interaction mediated by this notional particle between regions of different type, over *any* path. This answer to this question is the basis for our definition of Structural Entropy.

To simplify matters, let us suppose that each *R*_*i*_ has a distinct tag. This can be done without loss of generality because it is always possible to construct such a set. Let,

(6) $$R^{\prime }=\left\{⋃R_{i},\chi \left(R_{i}\right)=c,c\in C\right\}$$

where ⋃ denotes spatial union. *R*′ is a set of distinctly tagged regions.

We will model the trajectory of the particle as a Markov process. A Markov process (in discrete time) is characterised by a stochastic matrix, $Q=\left[q_{\mathrm{ij}}\right]$. Each element of this matrix, *q*_*ij*_, represents the probability that the notional particle, if it is in a region with the tag *c*_*i*_, will cross into a region with the tag *c*_*j*_ at the next time‐step.

The starting position of the particle is given by Eq. (5). That is, we assume that the particle has a chance to be starting in region *R*_*i*_ proportionally to its share of the volume. We write this distribution of starting positions as the column vector $p=\left[p_{i}\right]$. After one time‐step, the probability distribution of where the particle will be found is given by *Q* **p**. After *n* time‐steps, the distribution is given by *Q* ^*n*^ **p**. Using this, we can define the *n*th order Structural Entropy directly analogously to the *n*th order Path Entropy by,

(7) $$E_{n}=\left(Q^{n}p\right)\cdot \log\left(Q^{n}p\right)$$

where the notation $\log\left(x\right)$ for some vector $x=\left[x_{i}\right]$ means $\left[\log(x_{i})\right]$, and the product · is the standard vector dot‐ or inner‐product.

If the regions, *R*_*i*_ are connected, there is no partition in the graph of their adjacencies, there are no islands, then the Markov process described by *Q* is ergodic. A particle beginning in any region will eventually visit every other, and there will be a solution to the equation,

(8) π=Qπ

giving the unique stationary distribution *π* that is independent of the starting position (**p**; Pinsky & Karlin, 2010). We define the entropy of this distribution, if it exists, to be the Stationary Structural Entropy,

(9) $$E_{\pi }=\pi \cdot \log\left(\pi \right)$$

A method to calculate the *Q* remains to be determined. A principled way would be to say that a particle sufficiently close to a *R*_*i*_'s boundary has a chance of diffusing across the boundary into *R*_*j*_ proportionally to the fraction of *R*_*i*_'s total surface area that is adjacent to *R*_*j*_. Consider the figure on the right, showing the adjacency between *R*_1_ and *R*_2_. The shaded liminal region *δ* is taken to be the region where diffusion can happen. The liminal region is a buffer around of *R*_1_, extending outwards from the boundary, wherever there is an adjacent region. We can now work out the transition probabilities,
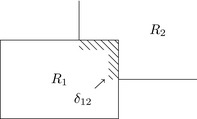

(10) $$q_{11}=\frac{\int_{R_{1}-\delta_{12}}dx}{\int_{R_{1}}dx}$$

(11) $$q_{12}=\frac{\int_{\delta_{12}}dx}{\int_{R_{1}}dx}$$

The chance to leave *R*_1_ for *R*_2_ is given by the fraction of *R*_1_'s volume that is near enough to *R*_2_ for the particle to diffuse across the boundary. The chance to remain in *R*_1_ is the fraction of its volume that is not sufficiently close to another region.

There are several reasonable ways to define the liminal region, *δ*. The most natural approach, suggested by the diagram, is for it to be the region within some constant distance of the boundary. This fails on practical grounds – namely, that determining the patch of the surface of *R*_1_ that is adjacent to *R*_2_ relies on the underlying data being sufficiently accurate and that there is a portion of their surfaces that are indeed spatially coincident. This is not actually the case in practice with the available data.

We work around this limitation of the data in the following way. We determine the portion of *R*_1_'s volume is near to *R*_2_ by dilating the latter by a small amount, *k*, and take the intersection of *R*_1_ and the dilated region, denoted by *D*(*R*_2_, *k*). We then calculate the transition probabilities by first calculating the relative volumes of a region and its liminal volumes with adjacent neighbours,

(12) $$v_{\mathrm{ij}}=\left\{\begin{array}{c} \int_{R_{i}\cap D\left(R_{j},k\right)}dxi\neq j\\ \int_{R_{i}}dx-\sum_{i\neq j}v_{\mathrm{ij}}\text{otherwise} \end{array}\right.$$

and then construct the transition probabilities by normalising,

(13) $$q_{\mathrm{ij}}=\frac{v_{\mathrm{ij}}}{\sum_{j}v_{\mathrm{ij}}}$$

## Results

### The Mouse Atlas

**Figure 2:**
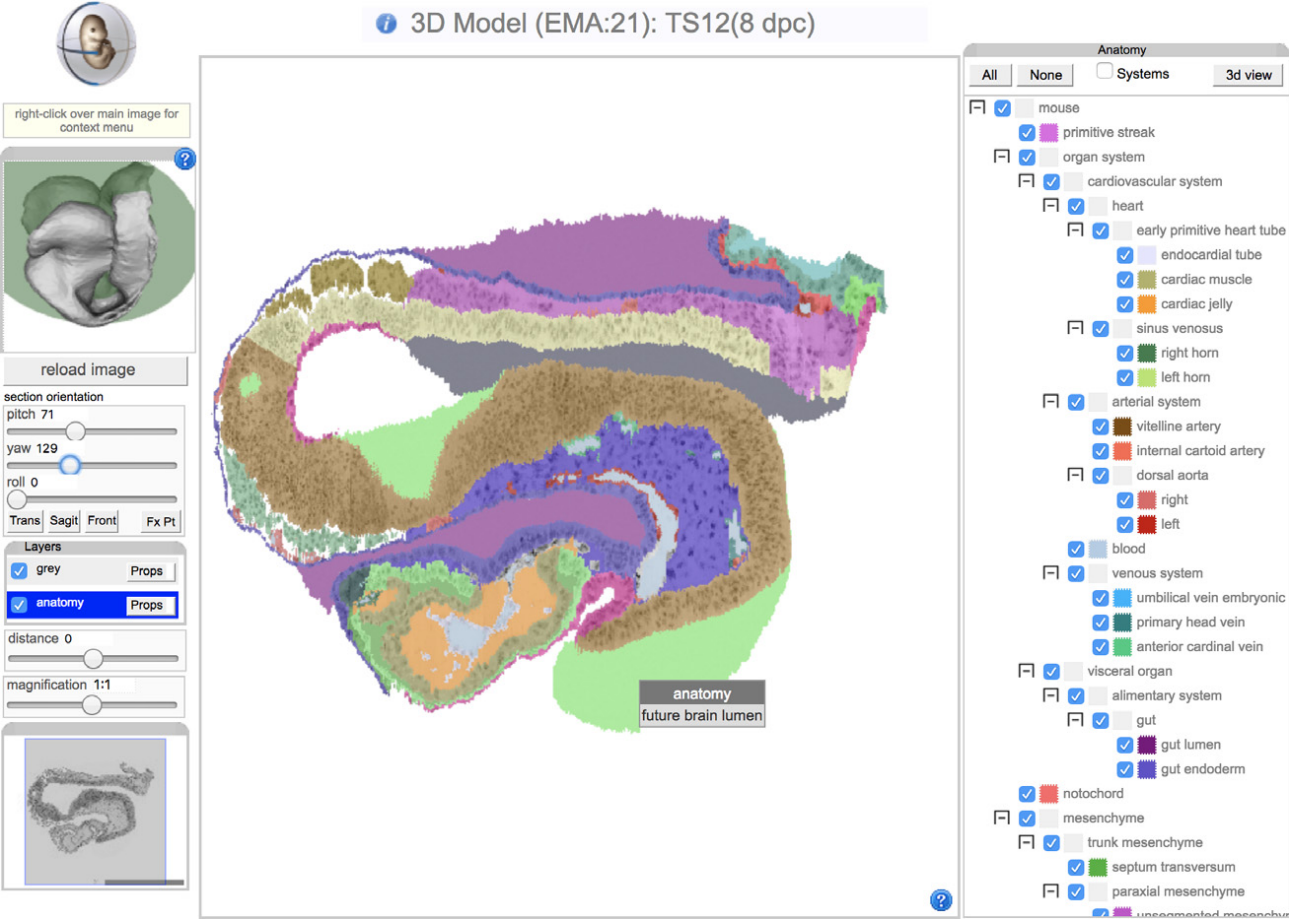
The on‐line eMouseAtlas viewer inspecting a cross‐section of the tagged embryo at Theiler stage 12.

The eMouseAtlas contains, in addition to genomic data and a large amount of structured metadata, 3D geometrical models of the delineated anatomy of mouse embryos at several stages of pre‐natal development. In total, there are 69 embryo models available to download (Armit et al., 2017) covering Theiler's morphological stages (Theiler, 1989) 7 through 26. Of these, the majority contain untagged 3D reconstructions and Optical Projection Tomography (OPT) images, but there are 22 with anatomy delineations (Fig. 2).

**Figure 3:**
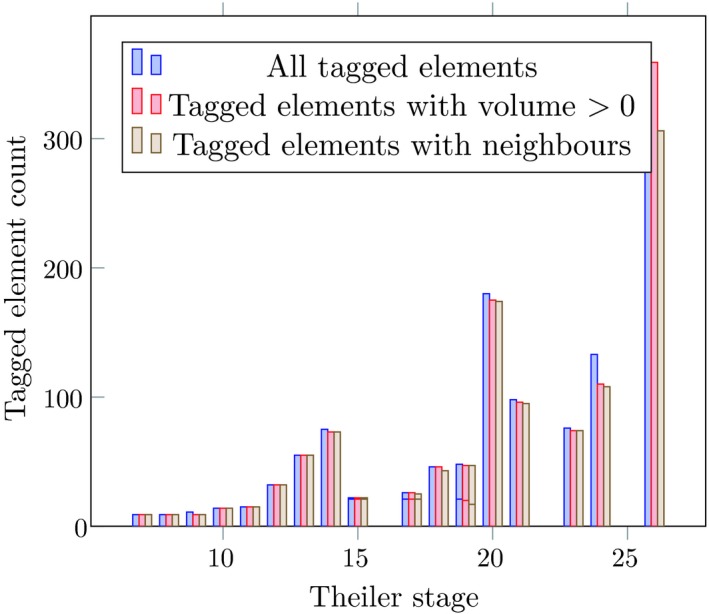
Basic statistics about datasets from the eMouseAtlas with anatomical delineations. Some datasets contain tagged elements with zero volume, or tagged elements which are not adjacent to any other. Elements with zero volume indicate a problem with the underlying data. For example ema27 at Theiler Stage 14 has zero volume elements for the left and right umbilical veins.

Figure 3 shows some basic information about the delineated datasets. Each 3D dataset is reconstructed (Hill & Baldock, 2015) from a series of 2D images arranged in layers. The datasets are made available in the Woolz format (Piper & Rutovitz, 1985) which is both compact and suitable for computation of spatial operations such as union, intersection, convex hulls, and so forth. We will be concerned with volumes of and adjacency relations between tagged elements, or in other words the sizes of anatomical regions and which are in physical contact with each other. For this reason, in addition to the count of tagged elements in each dataset, Fig. 3 shows counts of tagged geometrical elements with non‐zero volume and those that touch at least one other tagged element.

It is evident that something unexpected is happening in Fig. 3. It should not be the case that a mouse embryo loses anatomical diversity as it develops. The data for stages 15 through 19 and 21 through 25 seem particularly problematic. The explanation for this turns out to be quite mundane. The first stages were tagged manually, at significant cost, and resources were unfortunately not available consistently to continue this work (Baldock and Hill, pers. comm.). In some cases the latter stages appear to have been tagged according to the particular interest of the researcher doing the work. This bias in the data is nevertheless interesting in understanding how to interpret our complexity measure in terms of intrinsic or extrinsic structure, which we discuss further below. Despite these defects in the data, we are able to obtain a signal, albeit a noisy one.

We have excluded several models from the following analysis. Although ema149, at Theiler Stage 25, contains 78 delineated tissues, only four have non zero volume and only two have neighbours. Models ema76, ema103, ema108 and ema118 contain disconnected regions. This results in a *q*_*ij*_ that is not ergodic and therefore the Stationary Structural Entropy does not exist. Finally, ema36 is an outlier suggesting a drastically different tissue delineation methodology. Its statistics are reported but excluded from the figures.

### Structural Entropy of the eMouseAtlas

We now apply our Structural Entropy measure to the Mouse Atlas. Each stage has a different number of tagged elements. As our goal is to quantify the degree of structure, for each stage, we compare the Structural Entropy (Eqs (7) and (9)) to the maximum possible value given by,

(14) $$E_{\max}=-\log\left(\frac{1}{m}\right)$$

where *m* is the number of tagged elements. It is easy to see that as the number of tagged elements increases, the maximum entropy (degree of disorder) likewise increases. From this, we can define the normalised entropies,

(15) $${\overset{¯}{E}}_{\cdot }=\frac{E_{\cdot }}{E_{\max}}$$

which take on values from 0 to 1 and thus allow for comparison of the relative degree of disorder between developmental stages with different sets of tags. A value of 0 represents maximal structure, and 1 maximal disorder.

**Figure 4:**
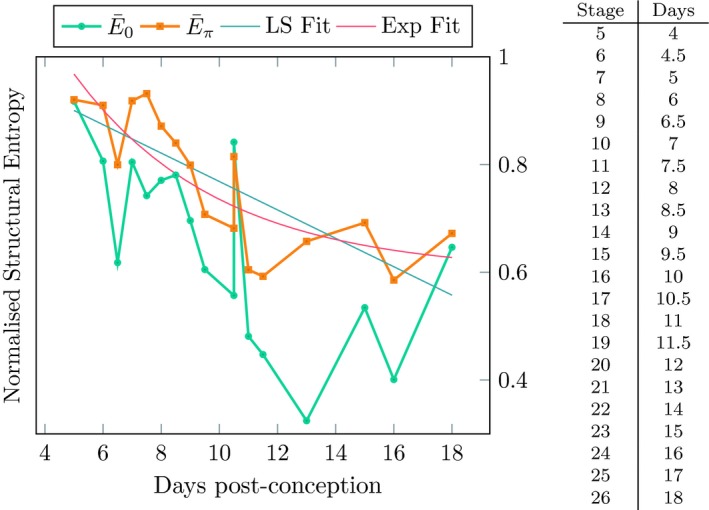
Normalised Structural Entropy as calculated for the eMouseAtlas data. Also shown is the least squares fit (LS Fit in the figure, with mean squared error 5.5 × 10^−3^) for the Stationary Structural Entropy and a trial exponential fit (Exp Fit in the figure, mean squared error 4.5 × 10^−3^). The table at right gives the correspondence between the Theiler stages present in the data and the time in days post‐conception. Excluded from this figure is, ${\overset{¯}{E}}_{0}$ (ema36) = 0.83, ${\overset{¯}{E}}_{\pi }$ (ema36) = 0.31.

The results of this calculation are presented in Fig. 4 and plotted against time measured in days post‐conception. Two curves are shown, one for ${\overset{¯}{E}}_{0}$, showing the amount of structure that is attributable purely to the volume distribution of tagged elements, with no account taken of their spatial relationships. The second curve, for ${\overset{¯}{E}}_{\pi }$, corresponds to the stationary distribution of the random walk among the tagged elements, as described above. The latter incorporates information about the volume through the *q*_*ii*_ as well as the spatial relationships through the *q*_*ij*_, *i* ≠ *j*.

In both cases, we see a decreasing trend. This is interpreted as a decrease in disorder, or an increase in structure, as the mouse embryo develops. This signal is much clearer in the case of ${\overset{¯}{E}}_{\pi }$, which displays an orderly, almost linear decrease. Indeed, a least squares fit for the normalised Stationary Structural Entropy has a mean squared error of 5.5 × 10^−3^, or two orders of magnitude smaller than the range of the entropy over the developmental phases covered by the dataset.

Clearly the decrease in disorder cannot be more than piece‐wise linear as that would imply the nonsensical result that at some stage the organism becomes perfectly ordered with exactly one tissue as *E*_·_ → 0 and beyond to negative values of entropy which defy interpretation. A trial exponential fit is also shown, ${\overset{¯}{E}}_{\text{fit}}=e^{-0.2t}+0.6$, that does not suffer from this problem of interpretation and has a mean squared error of 4.5 × 10^−3^.

The data at early developmental stages bear closer inspection. Although the general trend of our Stationary Structural Entropy measure, ${\overset{¯}{E}}_{\pi }$, is a steady decrease throughout the 13 days of development depicted in Fig. 4, there is a short period, from days 7 to 8 (Theiler stages 10–11), in which ${\overset{¯}{E}}_{\pi }$ rises before returning to the trend. This period corresponds to one of the most remarkable events of metazoan development, gastrulation, when the primitive streak forms and cell movements in and through the epiblast transform the relatively orderly bilaminar disc into the three germ layers of the body. Gastrulation is widely regarded as being pivotal in development, Lewis Wolpert famously remarking that it is a life event more important than birth and marriage. It is interesting that this special stage of embryogenesis is detected by our tracking Stationary Structural Entropy over time.

**Figure 5:**
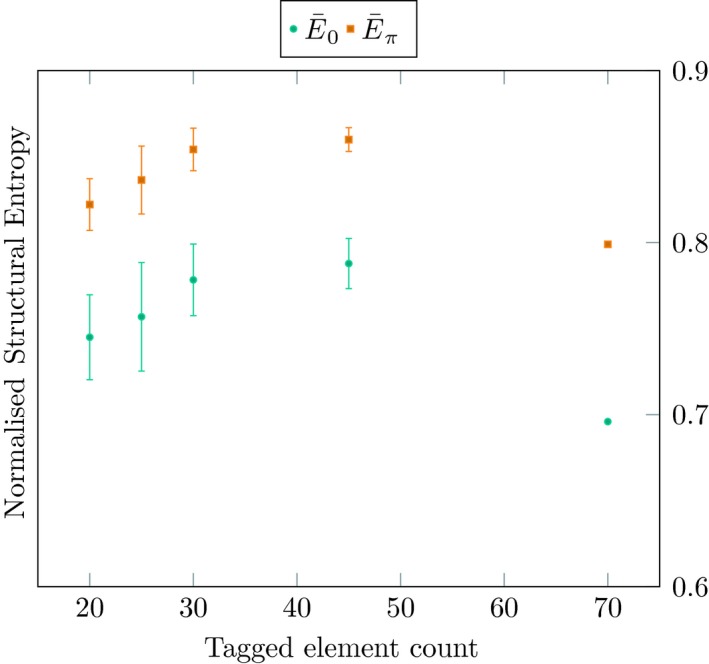
Normalised 0th order and Stationary Structural Entropy for models created by merging tagged elements from ema27, at Theiler stage 14. The data points isolated at the far right are for the original model. The merged models are created by merging at different depths: pairs, triples or quadruples of adjacent tissues. For each depth, 25 random models are generated and the resulting entropies are plotted according to the resulting number of tagged elements. The element count is discretised or grouped, e.g. 20–25 elements, 25–30 elements, and so forth. Error bars represent one standard deviation within a group.

To ascertain the extent to which the Structural Entropy calculation is biased by the number of tagged elements, we focus on a particular model, ema27 from Theiler stage 14. This model contains 75 tagged elements, of which 73 have non‐zero volume. To understand how the Structural Entropy changes as the number of elements decreases, we merge adjacent elements. We do this by iterating through the list of elements, and merging between one and four neighbouring elements, chosen at random. We then calculate the Structural Entropy and Stationary Structural Entropy on this merged model (Fig. 5).

We see that by randomly merging tagged elements, we introduce greater disorder. This is not unexpected. The original model was tagged in a particular way intended to correspond to an anatomical understanding of the embryo. This experiment takes no account of that, it simply merges elements that happen to be adjacent. With that done, both the Structural Entropy and the Stationary Structural Entropy are relatively stable with 30–60% of elements merged. Only when a clear majority of the elements are merged do these measures change appreciably. In particular, we find a correlation of entropy and element count between 0.2 and 0.3, suggesting only a weak correlation between our measure and the absolute number of tagged elements.

## Discussion

When Claude Shannon was discussing with John von Neumann what to call the quantity that came to be known as entropy in Information Theory, the latter famously quipped,

You should call it entropy, for two reasons. In the first place your uncertainty function has been used in statistical mechanics under that name, so it already has a name. In the second place, and more important, nobody knows what entropy really is, so in a debate you will always have the advantage. (Tribus & McIrvine, 1971)

In an important sense, information theoretic entropy is an attributed quantity. It is a measure, as Shannon originally called it, of *uncertainty* about the state of a system. The trick that we have performed here is to define such a system: a particle moving at random through the organs of an embryonic mouse. We then suggested that our uncertainty about the whereabouts of the particle corresponds in some way to the structural complexity of the organism itself. Tissues of different sizes contribute to our complexity measure in the following way. The measure is scale‐independent in the sense that absolute tissue size plays no role. Embryos containing a given number of tissues, all of the same size, will have the same Structural Entropy regardless of their size. If the tissue sizes are different, the Structural Entropy will be correspondingly smaller. The degree of difference is the essence of order, to a first approximation. This is captured by the 0th order measure, *E*_0_, describing the role played by tissue volume alone.

To account for the spatial arrangement of tissues, we incorporate information about the connectivity between tissues. When we consider geometrically complex structures, an important feature is that their surface area is large compared with their volume. This large surface area means that the liminal region, or region of connectivity with adjacent tissues, is also larger. This is the reason we claim that when we calculate the Stationary Structural Entropy, *E*_*π*_, it captures this kind of structural complexity. More complex tissues ‘communicate’ more with their neighbours and this, in turn, contributes to a decrease in the Stationary Structural Entropy. The relative difference between *E*_0_ and *E*_*π*_ encodes the amount of organisation that can be attributed to the spatial arrangements as opposed to simply the amount of matter.

This approach may or may not be reasonable. We believe that it is, mainly because it accords with our intuition about what such order or structure ought to mean. It captures the sense that, despite the proliferation of tissues as the embryo develops, the organism becomes more ordered. If it did not, it would simply be a jumble of cells, an upper bound on disorder such as measured by Davies (2016) using the taxonomy of cell types. That this is an upper bound is precisely what we see here: as development progresses, the Stationary Structural Entropy decreases relative to the equivalent disordered system, and it does so nearly consistently.

Another important aspect of the attributive nature of entropy arises from the data itself. In order to correctly compare like with like, each dataset should be tagged in the same way, using the same criteria. We have seen that there are defects in the data, with some datasets processed meticulously and some processed more coarsely. Even if the data were consistently and meticulously processed it could be argued that measures such as Structural Entropy say more about the complexity of the underlying theoretical anatomical model than the intrinsic complexity of the body of the mouse. We can, however, only work with the data and theoretical tools that we have. By deriving randomly merged models we can see that our Structural Entropy measure is only weakly dependent on the absolute number of tagged elements.

The potential application of Structural Entropy to neuroscience, ageing and psychological disorders appears promising. de Reus et al. (2014) considered the human brain connectome in an ‘edge‐centric’ as opposed to a ‘node‐centric’ way. In that article, communities of edges are identified; they seem to be significant but the meaning is left open: ‘The biological meaning of link communities in the brain is not immediately clear and very much open to scientific debate’. The distinction between edge‐centric and node‐centric is reminiscent of that between *E*_0_ and *E*_*π*_ above. De Reus’ approach was applied as a measure of brain structure as a baseline in healthy elderly populations (Perry et al., 2015). Yeo et al. (2016) suggest that de Reus’ approach may provide a useful indicator for psychological phenomena like schizophrenia, where differences were found, but it is unclear whether they are really significant or due to differences in methodology. There have also been some attempts to link it to general cognitive ability (Llufriu et al., 2017).

Voxel Based Morphometry (VBM; Ashburner & Friston, 2000) is now a standard technique for comparing magnetic resonance imaging (MRI) scans tagged in a similar way to the anatomical data that we have been considering. After some pre‐processing – tagging, smoothing and registering images to the same spatial coordinates – the scans are compared voxel‐wise. Among many applications, this approach has been famously used to show plasticity in response to environmental demands (Maguire et al., 2000), that grey matter normally decreases linearly with age (Good et al., 2001), and to ascertain the degree of progression of Alzheimer's disease (Testa et al., 2004; Matsuda, 2013). VBM shares some pre‐processing requirements with what we can call Structural Entropy Morphometry (SEM), but then proceeds very differently. VBM is a calculation on voxels (or pixels in two dimensions) and SEM is explicitly not, it is concerned with the geometry of the tagged elements themselves. Crucially, VBM measures the relationship of scans from different groups, whereas SEM is an intrinsic measure of the tagged object. Nevertheless it is plausible that SEM could recover the results of applying VBM and could yield additional insight. This possibility suggests potentially fruitful further research.

The concept of, and ways of method for measuring, structural entropy can be applied to a wider range of problems than normal embryonic development. Much research attention is currently being expended on developing organoids – small structures made from stem cells that are intended to capture enough of the essence of a natural organ to be useful for research (reviewed by Davies & Lawrence, 2018). There is much debate within that field about how faithfully organoids, particularly organoids made by the different techniques of different laboratories, capture the complexity of the organ they are intended to represent. Structural Entropy might be one useful measure. Another possible application is phylogeny: when discussing evolution, and particularly evolutionary developmental biology, it would be useful to have an objective measure of the anatomical complexity of adult organisms of different phyla or clades.

In this paper, we have called for the increased availability of high‐quality tagged 3D datasets for the development of computational tools for anatomy. We have examined the eMouseAtlas dataset and produced some basic statistics about the tagging and annotation. We have extended Path Entropy to account for spatial structure and introduced Structural Entropy and studied the stationary distribution of a particle's random walk through tagged anatomical regions of developing mouse embryos. The stationary distribution illustrates clearly how the organism becomes more spatially structured as it develops. Finally, applications of Structural Entropy morphometry to neuroscience and the study of diseases related to ageing have been suggested as areas for future research.

## Glossary

Edge: A connection between two vertices on a graph (qv).
Entropy: A measure of disorder: a highly ordered system (e.g. a perfectly alternating sequence of black and white tiles) has high entropy.
Graph: A mathematical structure used to model pairwise relationships between objects. Graphs consist of ‘vertices’ (the objects themselves) and ‘edges’ (lines that connect them). In a model of a random walk, for example, the vertices might represent the spatial location of each footprint and the edges of the strides that connect them.
Information Theory: A field of science that focuses on the quantification, storage, retrieval and communication of information, particularly with relation to entropy.
Tag: A tissue‐type annotation associated with a spatial point on a digital model of an embryo; e.g. point (99, 65, 432) might have the tag *bladder urothelium*. Note that in the section ‘Technical description of method’, the word ‘colour’ would usually be used in computer science or mathematics.
Vertex: An elementary object in a graph (qv).

## Authors’ contributions

W.W. and J.A.D. both contributed to the development of the concept of applying the *Path Entropy* measure to tagged anatomical models. W.W. extended the discrete formulation to apply to geometrical objects, implemented the data processing software. Both authors contributed equally to the main text.
